# *Clostridioides difficile* TcdB induces expression of its receptor (CSPG4) through a noncanonical Hippo signaling mechanism

**DOI:** 10.1016/j.jbc.2026.111137

**Published:** 2026-01-07

**Authors:** Jason L. Larabee, Elizabeth J. Donald, Anushka A. Sukhadia, Tyler M. Shadid, Sarah J. Miller, Jimmy D. Ballard

**Affiliations:** Department of Microbiology and Immunology, The University of Oklahoma Health Campus, Oklahoma City, Oklahoma, USA

**Keywords:** *Clostridioides difficile*, Chondroitin sulfate proteoglycan 4 (CSPG4), TcdB, Hippo signaling

## Abstract

Chondroitin sulfate proteoglycan 4 (CSPG4) is a major receptor for *Clostridioides difficile* TcdB, but the dynamics and regulation of CSPG4 expression during *C*. *difficile* disease has not been described. Using a combination of experimental approaches, we discovered that TcdB induces CSPG4 expression through a mechanism involving small GTPase inactivation and modulation of kinases in the Hippo-signaling cascade. Treatment of HeLa cells or human pericytes with TcdB increased CSPG4 expression, and this could be mimicked by chemical inhibition of Rho. Experiments further demonstrated that TcdB-induced expression of CSPG4 is blocked by inhibitors of two core Hippo kinases (MST1/2 and LATS1/2), but the typical downstream target (YAP/TAZ) of these regulators was not required for the changes in CSPG4. Instead, data from RNA-seq and CUT&RUN experiments found CSPG4 expression was modulated by CCCTC-binding factor (CTCF), a lesser-known target of Hippo signaling. CTCF is a DNA-binding protein capable of repressing gene transcription, and our work found that reduced CTCF leads to increased CSPG4 expression. Additionally, CTCF binding at the *CSPG4* gene locus is eliminated by TcdB activity. These data support a model in which TcdB upregulates CSPG4 *via* Rho inactivation and subsequent Hippo-mediated inactivation of the transcriptional repressor CTCF.

Chondroitin sulfate proteoglycan-4 (CSPG4) is a glycosylated cell surface protein found on a subset of mammalian cells (1). This transmembrane protein is involved in cell adhesion, migration, and growth, and its hyperexpression is associated with cancers such as melanoma and glioblastoma (2, 3, 4, 5, 6). CSPG4 is also targeted by TcdB, a bacterial toxin produced by *Clostridioides difficile* (7). While distinct variants of TcdB exist, major variants such as TcdB1 and TcdB2 utilize CSPG4 as a primary receptor for entering target cells (8). Despite its broad importance in cell function, cancer, and bacterial infection, only a limited number of studies have explored the mechanisms regulating the expression of CSPG4 (9, 10). Our work here provides additional insight into the mechanisms regulating CSPG4 expression.

In previous work designed to identify factors involved in cellular sensitivity to TcdB, we passaged cells in the presence of increasing amounts of TcdB and enriched for cells that gained resistance to the toxin (10). A primary mechanism of TcdB resistance discovered in the earlier study was loss of CSPG4 expression, and transcriptional profiling correlated this with alterations in the Hippo signaling pathway (10). This work revealed a direct connection between Hippo signaling and expression of CSPG4. Modulating Hippo signaling *in vivo* reduced levels of CSPG4 in the intestines and protected mice from *C*. *difficile* infection, presumably due to the absence of TcdB mediated cytotoxicity (10).

The discovery that Hippo signaling regulates CSPG4 suggested an unexplored intersection between TcdB activity, Hippo signaling, and the expression of CSPG4. Following cell entry, TcdB inactivates small GTPases, such as Rho, that were previously found to activate the Hippo signaling pathway (11). Thus, in theory the intracellular activity of TcdB could modulate expression of its own receptor through the Rho/Hippo/CSPG4 axis. In the current study, we dissected the relationship between TcdB and the regulation of CSPG4 expression by Hippo signaling. The data revealed TcdB upregulates CSPG4 through a noncanonical Hippo signaling mechanism. These findings provide insights into both CSPG4 regulation and a phenomenon wherein an intracellular bacterial toxin drives expression of its own cellular receptor.

## Results

### Exposure to *C*. *difficile* TcdB increases cellular CSPG4

**Figure 1:**
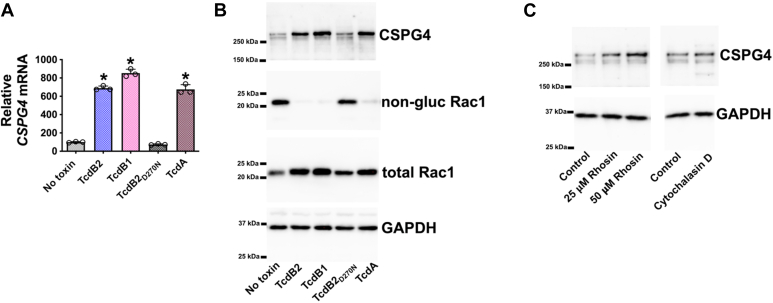
**CSPG4 levels increase following exposure to *C*. *difficile* toxins**. *A*, HeLa cells were exposed to 1 ng/ml of toxin for 24 h, and multiple wells from each treatment group were combined for RNA isolation. RT-qPCR was used to quantify *CSPG4* transcripts. RT-qPCR data are presented as mean (n = 3) ± S.D with each data point as a technical replicate. ∗*p* < 0.05 comparing no toxin cells and toxin-treated cells using Student’s *t* test. *B*, immunoblot from protein lysates acquired from HeLa cells exposed to 1 ng/ml of toxin for 24 h. *C*, immunoblot from protein lysates prepared from HeLa cells exposed for 24 h to Rhosin (25 μM and 50 μM) or cytochalasin D (10 μM).

As shown in Figure 1, HeLa cells were exposed to TcdB and then changes in CSPG4 expression were determined at the transcript and protein level. Results of this work revealed that TcdB1 and TcdB2 are each able to increase levels of the CSPG4 transcript and protein (Fig. 1, *A* and *B*). TcdB1 and TcdB2 are highly homologous and disease-relevant forms of TcdB. Both TcdB1 and TcdB2 use CSPG4 as their primary receptor (8); however, TcdB1 also binds frizzled proteins (FZD1,2,7) while TcdB2 does not (12). The mechanism of action used by both forms of TcdB involves this toxin entering host cells and glucosylating a critical threonine on small GTPases (Rho, Rac, and Cdc42). For these studies, glucosylation was confirmed using an antibody that reacts only with nonglucosylated Rac1 (Fig. 1*B*). To test if the glucosylation activity of TcdB is necessary to induce CSPG4 expression, experiments were conducted utilizing a mutant form of TcdB (TcdB2_D270N_) that enters cells but lacks the enzymatic activity required to glucosylate substrates. As shown in Figure 1, *A* and *B*, TcdB2_D270N_ does not increase CSPG4 at either the transcript or protein level and, as expected, does not glucosylate Rac1. We also determined if CSPG4 expression is induced by TcdA, which is a large clostridial toxin that glucosylates a similar subset of small GTPases as TcdB but enters cells *via* a separate mechanism. Similar to TcdB, TcdA induced the expression of CSPG4, further demonstrating that inactivation of small GTPases correlates with the upregulation of CSPG4 (Fig. 1, *A* and *B*).

Hippo signaling can be modulated by mechanosensing mechanisms, including those related to changes in actin polymerization (13). Since TcdB activity results in the disruption of the cytoskeleton through the deactivation of small GTPases (Rho, Rac, and Cdc42), our next experiment determined whether TcdB increases CSPG4 through a mechanism depending on the collapse of the cytoskeleton or through the direct inactivation of GTPases. We tested whether CSPG4 is increased when the cytoskeleton is disrupted using cytochalasin D, which is a small molecule that disrupts the cytoskeleton through a non-GTPase mechanism (14). As shown in Figure 1*C*, treatment with cytochalasin D did not alter levels of detectable CSPG4. In contrast, treatment of cells with Rhosin, a small molecule inhibitor specific for Rho, resulted in an increase in CSPG4 (Fig. 1*C*).

**Figure 2:**
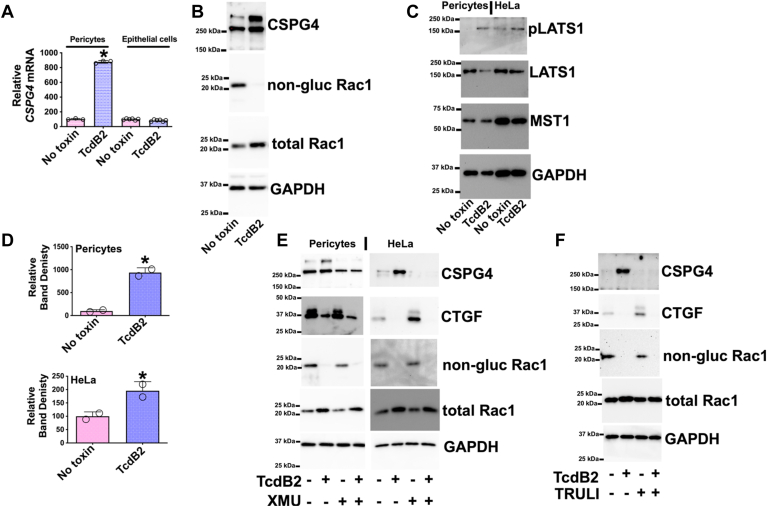
**Core Hippo kinase regulation of CSPG4 in TcdB exposed pericytes**. *A*, pericytes were exposed to 1 ng/ml of TcdB2 for 24 h and colonic epithelial cells were exposed to 10 ng/ml of TcdB2 for 24 h. Multiple wells from each treatment were combined for RNA isolation. RT-qPCR was used to quantify *CSPG4* transcripts. RT-qPCR data are presented as mean (n = 3) ± S.D. with each data point as a technical replicate. ∗*p* < 0.05 determined by Student’s *t* test. *B*, Immunoblots from protein lysates acquired from pericytes exposed for 24 h to 1 ng/ml of TcdB. *C*, immunoblots from protein lysates obtained from pericytes or HeLa cells exposed for 24 h to 1 ng/ml of TcdB. Phosphorylated LATS1 (pLATS1) was detected with an antibody recognizing phosphorylation at Thr 1079. *D*, densitometry analysis of pLATS1 immunoblots. Relative band density is presented as mean (n = 2) ± S.D. with each data point as a technical replicate. ∗*p* < 0.05 determined by Student’s *t* test. *E*, immunoblots from protein lysates acquired from pericyte or HeLa cells exposed for 24 h to 1 ng/ml of TcdB in the presence or absence of 10 μM XMU-MP-1. *F*, immunoblots from protein lysates taken from HeLa cells exposed for 24 h to 1 ng/ml of TcdB with and without of 30 μM TRULI.

Because a recent study suggested pericytes are targeted by TcdB during *C*. *difficile* infection (15), these cells were also evaluated to determine if toxin induces CSPG4. Pericytes are contractile cells that wrap around the subepithelial blood vessels in the colonic mucosa and are the major CSPG4 expressing cells in the colon (16). For these experiments, primary human pericytes were cultured and exposed to TcdB2. Similar to HeLa cells, results revealed TcdB2 increased CSPG4 at both the transcript and protein level (Fig. 2, *A* and *B*). In contrast, expression of *CSPG4* was not detected or induced by TcdB2 in cultured primary human colonic epithelial cells (Fig. 2*A*).

### TcdB induces CSPG4 through activation of core Hippo kinases

**Figure 3:**
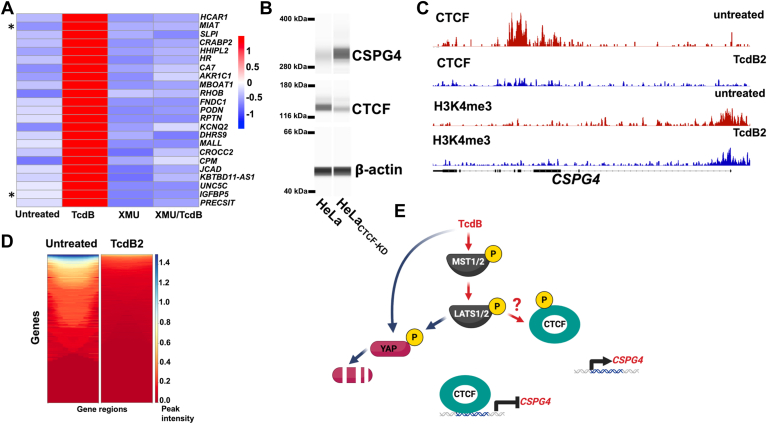
**CSPG4 is upregulated by TcdB independent of YAP**. *A*, immunoblots from protein lysates acquired from pericytes exposed for 24 h to 1 ng/ml of TcdB and/or 10 μM XMU-MP-1. *B*, heatmaps depicting Z-score values for YAP regulated genes generated from RNA-seq analysis of pericytes exposed for 24 h to 1 ng/ml of TcdB and/or 10 μM XMU-MP-1. *C*, heatmaps showing Z-score values for MTF1 regulated genes generated from RNA-seq analysis of pericytes exposed for 24 h to 1 ng/ml of TcdB and/or 10 μM XMU-MP-1. *D*, immunoblots from protein lysates taken from HeLa cells transfected for 48 h with control plasmid (pCMV-GFP) or a plasmid for expressing YAP_S5A_ (pCMV-flag YAP2 5SA). For the final 24 h, the transfected cells were exposed to 1 ng/ml of TcdB.

Our previous work demonstrated that inactivation of Hippo signaling downregulates CSPG4 expression (10), and studies by another group have demonstrated that TcdB inactivates the primary transcriptional regulators of Hippo signaling (YAP/TAZ) (17). These data suggest that Hippo signaling may be necessary for TcdB to upregulate CSPG4; thus, we investigated this possibility by first determining whether TcdB activates the core Hippo kinases (*i*.*e*. MST1/2 and LATS1/2; see Figure 3*E* for pathway representation). To determine the activity of the core Hippo kinases following TcdB treatment, we examined phosphorylation of the Thr 1079 residue in LATS1, which is a target of the MST1 kinase when Hippo signaling is activated (18). As shown by immunoblot in Figure 2, *C* and *D*, TcdB2 treatment of either pericytes or HeLa cells elevated phosphorylation of Thr 1079 in LATS1. In HeLa cells, this increase in LATS1 phosphorylation occurred without TcdB2 changing levels of total LATS1 (Fig. 2*C*). However, in pericytes, TcdB-mediated phosphorylation of LATS1 occurred with a concomitant decrease in total LATS1 (Fig. 2*C*), likely the result of a feedback loop in the Hippo pathway similar to that described in other studies (19, 20).

**Figure 4:**
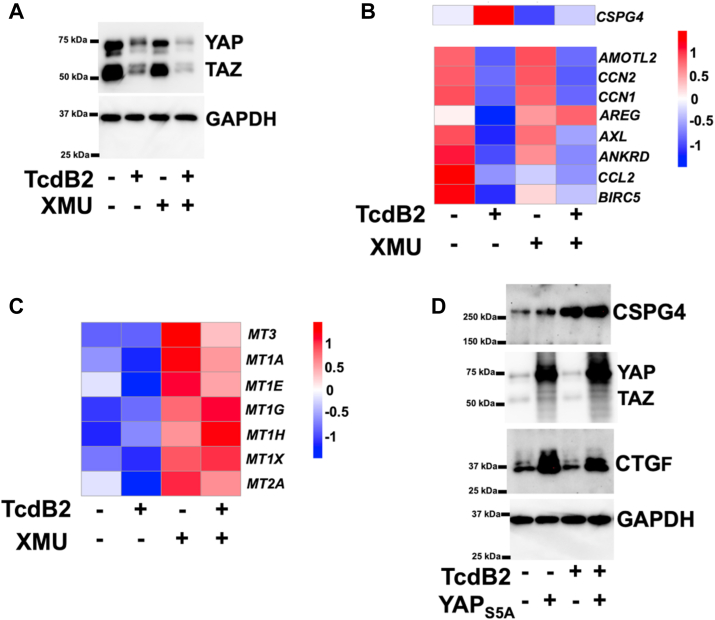
**CSPG4 is regulated by CTCF**. *A*, heatmaps representing Z-score values for the top TcdB induced genes that significantly differ from the TcdB + XMU-MP-1 group. These data are obtained from RNA-seq analysis of pericytes exposed for 24 h to 1 ng/ml of TcdB and/or 10 μM XMU-MP-1. *B*, representative bands from a capillary separation immunodetection system obtained from protein lysates generated from HeLa cells and HeLa_CTCF-KD_. *C*, genome browser tracks displaying CUT&RUN experiment for HeLa cells left untreated or exposed to 1 ng/ml of TcdB2 for 24 h. *D*, heatmaps displaying CTCF changes in DNA binding throughout the genome. *E*, model describing how TcdB modulates Hippo signaling and increases CSPG4.

Because TcdB appears to activate core Hippo kinases, we next determined if inhibiting these kinases prevents TcdB-mediated increases in CSPG4. First, we tested XMU-MP-1, which inhibits the activity of MST1/2 (21) and was previously used to prevent *C*. *difficile* disease in a mouse model (10). As shown in Figure 2*E* by immunoblot, treating pericytes or HeLa cells with XMU-MP-1 prevented the TcdB2-mediated increase in CSPG4. A similar analysis was performed using TRULI, an inhibitor that targets LATS1/2 (22). As with XMU-MP-1, the LATS1/2 inhibitor prevented TcdB2 from promoting an increase in CSPG4 in HeLa cells (Fig. 2*F*). Both inhibitors blocked toxin-induced CSPG4 expression without disrupting the ability of TcdB to glucosylate intracellular Rac1 (Fig. 2, *E* and *F*). As expected, treatment with either inhibitor resulted in the upregulation of connective tissue growth factor (CTGF), a gene product known to be increased when Hippo signaling is inactivated (Fig. 2, *E* and *F*). Unexpectedly, neither drug overcame TcdB2-induced suppression of CTGF, suggesting noncanonical regulation of Hippo signaling by TcdB.

### Noncanonical regulation of Hippo signaling by TcdB

Because inactivation of Hippo signaling prevents TcdB from inducing CSPG4, we next investigated how TcdB modifies Hippo signaling in pericytes downstream of the core Hippo kinases. These experiments focused on the paralogs YAP and TAZ because these transcriptional regulators control the expression of many of the known Hippo-regulated genes and are degraded when Hippo signaling is activated. As shown by immunoblot in Figure 4*A*, levels of YAP and TAZ were evaluated in pericytes after TcdB2 treatment in the presence or absence of XMU-MP-1. The results demonstrated that cellular intoxication with TcdB2 reduces levels of YAP and TAZ; however, this effect is not reversed by the inhibitory effects of XMU-MP-1. These data further suggested TcdB-mediated inactivation of Rho modulates Hippo signaling in a non-canonical manner.

To gain a more comprehensive profile of how TcdB and XMU-MP-1 modify gene expression in pericytes, RNA-seq was carried out and the resulting gene expression profiles were analyzed focusing on *CSPG4* and a panel of genes that are regulated by YAP and TAZ. As shown in the heatmap in Figure 4*B*, TcdB2 exposures upregulated *CSPG4* while YAP/TAZ regulated genes (*AMOTL2*, *CCN2*, *CNN1*, *AREG*, *AXL*, *ANKRD*, *CCL2*, and *BIRC*5) were repressed. Treating pericytes with XMU-MP-1 blocked toxin-induced CSPG4 expression but did little to prevent toxin repression of YAP/TAZ target genes, with the exception of the *AREG* transcript (Fig. 4*B*). To verify XMU-MP-1 is effectively inhibiting the core Hippo kinases in pericytes, gene expression profiles were examined for the alternative LATS1/2 substrate MTF1 (metal regulatory transcription factor 1) (23). MTF1 is a transcription factor controlling cellular heavy metal responses and is inactivated by LATS1/2 phosphorylation (23); therefore, inhibition of core Hippo kinases should activate MTF1. As shown in a heatmap comprised of MTF1 target genes (Fig. 4*C*), TcdB2 repressed several MTF1-regulated genes while pericytes treated with XMU-MP-1 demonstrate a gene expression signature indicative of MTF1 activation even in the presence of TcdB2. Therefore, XMU-MP-1 inactivated core Hippo kinases as expected but did not overcome the toxin blockade on the canonical factors YAP and TAZ.

The core Hippo kinases regulate toxin-induced CSPG4 expression, but the data above indicate inhibition of the core Hippo kinases modestly restores toxin repression of YAP target genes. In the next set of experiments, we directly modulated YAP to determine if these factors mediate toxin-induced CSPG4 upregulation. In this experiment, HeLa cells were transfected with a YAP_S5A_ expression plasmid or a control plasmid, and then we determined if toxin induced CSPG4 could be blocked in the presence of this YAP mutant. YAP_S5A_ is a constitutively active YAP mutant not subject to regulation by core Hippo kinases (24). As shown by immunoblot in Figure 4*D*, transfection of HeLa cells with the YAP_S5A_ expression plasmid resulted in a high level of YAP_S5A_ expression and a concomitant increase in CTGF. As predicted, toxin exposure did not reduce YAP_S5A_ levels or reduce CTGF expression (Fig. 4*D*). Interestingly, expression of YAP_S5A_ did not alter the ability of TcdB2 to induce CSPG4, indicating that inactivating YAP is not necessary for TcdB to increase CSPG4.

### CTCF modulates CSPG4 levels

The data in Figure 4 indicate CSPG4 is induced by TcdB through a Hippo signaling mechanism independent of YAP and TAZ. This finding led us to consider other factors that are regulated by core Hippo kinases, such as CTCF (CCCTC-binding factor) (25, 26). CTCF is a DNA-binding protein that can act as a transcriptional repressor. Recent studies have found that the Hippo kinase LATS1/2 phosphorylates CTCF, and this blocks CTCF binding to DNA (25, 27). Therefore, we reasoned that TcdB-activated Hippo signaling could disrupt CTCF DNA binding and transcriptional repression. As a result, *CSPG4* expression should increase in the absence of transcriptional repression by CTCF. To determine if CTCF plays a role in TcdB-mediated gene expression, we first analyzed our transcriptome data from pericytes in order to determine if known CTCF responsive genes were increased by TcdB2 and then repressed by inhibition of Hippo signaling with XMU-MP-1. The top TcdB2 induced genes most significantly reversed by XMU-MP-1 are presented as a heatmap in Figure 3*A*. Examination of this list revealed two genes (*MIAT* and *IGFBP5*) that previous studies have found to be induced by loss of CTCF (28, 29).

As a result of the observations from the transcriptional profiles, our next experiments tested the hypothesis that TcdB modifies CTCF activity resulting in elevated CSPG4 expression. First, we ascertained if reducing CTCF would increase CSPG4 levels. For this experiment, CRISPR/Cas9 gene editing was used to generate a knock down cell line with reduced levels of *CTCF* (HeLa_CTCF-KD_) as shown in Figure 3*B*. A comparative analysis between HeLa_CTCF-KD_ and parental HeLa cells confirmed that CSPG4 levels increased when CTCF levels were reduced (Fig. 3*B*). Next, we determined whether CTCF-binding patterns at the *CSPG4* gene locus are altered by cellular intoxication with TcdB. For these experiments, CTCF binding was mapped using a CUT&RUN approach. As shown in the genome tracks in Figure 3*C*, untreated HeLa cells displayed CTCF binding to the *CSPG4* gene throughout exon 4 and 5. In HeLa cells exposed to TcdB2, the genome tracks demonstrated loss of CTCF binding at the *CSPG4* gene (Fig. 3*C*), while the tri-methylation status of histone H3 on Lys 4 remained near the start site of the *CSPG4* gene after TcdB2 treatment (Fig. 3*C*). Expanding our analysis to a genome-wide scale revealed loss of CTCF binding throughout the genome in TcdB2 treated HeLa cells, as shown in the heatmaps in Figure 3*D*.

As summarized in Figure 3*E*, our overall findings indicate TcdB upregulates CSPG4 through a noncanonical Hippo signaling mechanism. This mechanism depends on the core Hippo kinases but does not rely on the canonical transcriptional modifiers YAP/TAZ. Evaluation of other factors targeted by Hippo kinases revealed CTCF regulation of CSPG4 expression.

## Discussion

CSPG4 is required for TcdB entry into host cells and consequently *C*. *difficile* pathogenesis (8). As such, changes in CSPG4 expression in the colon could be a critical factor influencing whether or not the host experiences asymptomatic colonization or severe colitis. In these studies, we found that TcdB upregulates the expression of CSPG4 through a nonconical Hippo signaling mechanism in pericytes. Pericytes were examined in this study because these cells are the primary CSPG4 expressing cells in the colon and are putative target cells for TcdB during disease (15). During early stages of infection, the ability of TcdB to upregulate CSPG4 in pericytes could enhance the sensitivity of pericytes to TcdB or perhaps, more intriguingly, increase the amount of CSPG4 shed from these cells. Recent studies by Childress *et al*. have found that soluble CSPG4 facilitates TcdB activity on epithelial cells (30); therefore, increased CSPG4 expression in pericytes could lead to elevated soluble CSPG4 that expands TcdB tropism. Our results also indicate intoxication with TcdA upregulates CSPG4 expression, suggesting that less cytotoxic TcdA could enhance TcdB sensitivity. A similar effect was previously reported for anthrax toxin (edema toxin and lethal toxin) when edema toxin was found to increase the expression of anthrax toxin receptors (ANTXR1 and ANTXR2) (31), thus heightening sensitivity to lethal toxin.

A key finding of these studies is that TcdB induced CSPG4 expression through a Hippo signaling mechanism. Investigating this mechanism led to the conclusion that TcdB activates the core Hippo kinases (MST1/2 and LATS1/2) and the activation of these kinases is required for TcdB to upregulate CSPG4 (Fig. 2). Hippo signaling typically modifies gene expression through the activities of the transcriptional modifiers YAP/TAZ, and our previous studies indicated knocking out YAP leads to increased CSPG4 (10). However, these current studies revealed that toxin-induced CSPG4 expression does not utilize a mechanism that requires YAP/TAZ (Fig. 4). This notion is supported by some key observations. First, inhibition of MST1/2 or LATS1/2 does not prevent TcdB inactivation of YAP/TAZ. Second, inhibition of these kinases clearly modulates YAP/TAZ (Fig. 2, *E* and *F*) and alternative downstream LATS1/2 targets such as MTF1 (Fig. 4*C*). Third, TcdB induction of CSPG4 is not decreased by the expression of YAP_S5A_, which is not subject to regulation by core Hippo kinases (Fig. 4*D*). Therefore, TcdB is likely modulating YAP/TAZ through a mechanism independent of the core Hippo kinases. Indeed, there is precedent for this, as previous studies showed Rho signaling can regulate YAP through mechanisms that are both dependent and independent of the core Hippo kinases (11). Despite the focus on CSPG4, it is also important to note that the ability of *C*. *difficile* toxins to reduce YAP/TAZ activity could be critical to the etiology of *C*. *difficile* disease beyond receptor regulation since YAP/TAZ control pathways necessary for immunity, inflammation and tissue repair mechanisms (32).

CTCF is a DNA-binding protein that regulates gene transcription by acting as a repressor, an activator, or an insulator protein (33). Recent studies have found that the Hippo kinase LATS1/2 phosphorylates CTCF, resulting in the disruption of DNA-binding and changes in gene expression (25, 26). These findings paired with our observations eliminating YAP/TAZ as the primary downstream targets in TcdB-induced receptor expression, led us to explore CTCF’s potential role in more detail. Our work demonstrates that CTCF binds the *CSPG4* locus starting at intron 3 and spanning exons 4 and 5 (Fig. 3*C*), which agrees with data in the ChIP-Atlas taken from multiple cell types (https://chip-atlas.org) (34, 35, 36). Strikingly, the addition of TcdB abolished CTCF binding at the *CSPG4* locus, suggesting loss of CTCF repressor activity contributes to TcdB-mediated upregulation of CSPG4 (Fig. 3*C*). The ability of CTCF to repress CSPG4 is also supported by our data showing that knocking down CTCF expression results in CSPG4 upregulation (Fig. 3*B*).

Examination of CTCF binding throughout the genome revealed TcdB causes genome-wide loss of CTCF binding (Fig. 3*D*). CTCF is a global transcriptional regulator that can dramatically shift gene expression profiles (33), thus TcdB impacting CTCF would be expected to substantially alter gene expression. Indeed, examining the transcriptome of pericytes revealed that TcdB treatment causes a plethora of transcriptional changes with ∼ 320 transcripts significantly changed. Collectively, these results broaden the model for TcdB intoxication and provide a mechanistic understanding of the relationship between Rho inactivation, Hippo signaling, CTCF modulation, and upregulation of CSPG4.

## Experimental procedures

### Reagents and recombinant toxins

The following reagents were purchased from Sigma: XMU-MP-1 (product # SML2233) and cytochalasin D (C2618). TRULI (product # HY-138489) was purchased from MedChemExpress. Rhosin (5003) was obtained from Tocris Bioscience. TcdB1, TcdB2, TcdB2_D270N_ and TcdA were expressed and purified in a *Bacillus megaterium* recombinant system as previously described (37).

### Cell culture

HeLa cells (CCL-2) were purchased from American Type Culture Collection and cultured in Eagle's Minimum Essential Medium supplemented with 10% FBS, 100 units/ml penicillin, and 100 μg/ml streptomycin. Primary human brain pericytes (ACBRI 498) were purchased from Cell Systems and cultured following the protocol provided by Cell Systems. Primary human colonic epithelial cells (36,037–08) were obtained from Celprogen and were cultured using the protocol provided by Celprogen. All cells were grown at 37 °C in the presence of 5% CO_2_.

### Immunoassays

Cell lysates prepared by removing media, washing cells with PBS, and then adding 4° C lysis buffer containing 1% SDS, 50 mM Tris (pH 7.4), 5 mM EDTA, and Halt Protease Inhibitor Cocktail. Proteins within the lysates were then detected by immunoblot or in a capillary separation immunodetection system (Jess system; Bio-Techne). Immunoblots were developed using the Clarity Western Enhanced Chemiluminescence (ECL) Substrate (BIO-RAD) and images were captured utilizing a ChemiDoc MP Imaging System (BIO-RAD). For phosphorylated LATS1, band intensities were quantified using ImageJ (Fiji) and were normalized to total LATS1. Primary antibodies used were a mouse monoclonal antibody recognizing nonglucosylated Rac1 (BD Bioscience; catalog no. 610651); a mouse monoclonal antibody recognizing total Rac1 (EMD Millipore; catalog no. 05–389); a mouse monoclonal antibody against GAPDH (Abcam; product # ab8245); a mouse monoclonal against YAP/TAZ (Santa Cruz Biotechnology; product # sc-101199); a mouse monoclonal against MST1 (Santa Cruz Biotechnology; product # sc-515051); a rabbit monoclonal antibody against CSPG4 (Abcam; ab275024); a rabbit monoclonal antibody against ß-actin (Cell Signaling Technology; product # 4970); a rabbit monoclonal antibody against CTCF (Cell Signaling Technology; product # 3418); a rabbit monoclonal against LATS1 (Cell Signaling Technology; product #3477) a rabbit monoclonal against phosphorylated LATS1 (Thr1079) (Cell Signaling Technology; product #8654); and a rabbit monoclonal antibody against CTGF (Cell Signaling Technology; product # 86641).

### RT-qPCR

RNA was isolated from cultured cells using Direct-Zol microprep kits (Zymogen) and suspended in RNase-free water. Isolated RNA was then converted to cDNA in reverse transcription reactions using SuperScript IV VILO Master Mix (Invitrogen). The resulting cDNA was combined with Luna Universal qPCR Master Mix (New England Biolabs) and gene-specific primers. Amplification reactions were then performed with an Applied Biosystems QuantStudio 5 real-time PCR system. Relative changes in levels of the mRNA of the gene of interest were compared with the levels of human *ACTB* mRNA using the 2^−ΔΔCt^ method.

### RNA-Seq

RNA was isolated in duplicate from cultured pericytes using Direct-Zol microprep (Zymogen) and suspended in RNase-free water. Overall RNA purity and concentration was measured using a NanoDrop (ThermoFisher) and an Agilent Bioanalyzer (Agilent Technology). Stranded RNA-seq libraries were constructed with 1 μg of RNA using the NEBNext poly(A) mRNA isolation kit (New England Biolabs) followed directly by the XGen Broad Range RNA Library Prep kit (IDT). Each of the libraries was indexed during library construction to multiplex for sequencing. Libraries were quantified using a Qubit 4 fluorometer (Invitrogen) and checked for size and quality on an Agilent Bioanalyzer (Agilent Technology). Samples were normalized and pooled onto a 150 paired-end run on Illumina’s NextSeq 2000 Platform to obtain 50 M reads per sample.

The FASTQ files were processed and aligned to the Human GRCh38 genome using DRAGEN RNA v4.2.4 (Basespace Illumina). Differential expression was performed using DRAGEN Differential Expression v4.2.4 (Basespace Illumina). Output files generated from these analyses were used to produce heatmaps comparing four conditions (untreated, TcdB2, XMU-MP-1, and TcdB2/XMU-MP-1). To generate heatmaps, the DESeq2 package in R was used to create a normalized count matrix for all four conditions and a matrix containing all the genes significantly changed between the TcdB2 condition and the TcdB2/XMU-MP-1 condition. After combining these matrices and sorting based on the TcdB2 condition, heatmaps were created using the pheatmap package in R. For heatmaps containing specific sets of genes, the normalized count matrix for all four conditions was combined with a matrix containing specific genes.

### Transfection of cells

HeLa cells were transfected with the plasmid pCMV-flag YAP2 5SA, which was a gift from Kunliang Guan (Addgene plasmid # 27371; http://n2t.net/addgene:27371) (24). HeLa cells were also transfected with the control plasmid pCMV-GFP, which was a gift from Connie Cepko (Addgene plasmid # 11153; http://n2t.net/addgene:11153) (38). To transfect HeLa cells, the cells were plated at 1 x 10^5^ cells per well of a 12 well tissue culture plate in EMEM with 10% FBS and allowed to grow overnight. The following day, a transfection mixture containing 4 μl of FuGENE HD (Promega) and 1 μg of plasmid DNA (4:1 ratio of FuGENE HD to DNA) was prepared in 50 μl tissue-culture media (free of FBS and antibiotic). This was then added to one well of a 12 well tissue culture plate containing the HeLa cells cultured in EMEM with 10% FBS. After a 5 h exposure, the media was removed and replaced with fresh media.

### Generation of CTCF knockdown

HeLa cells were transfected with a mixture of two CTCF double nickase plasmids (Santa Cruz Biotechnology; sc-401245-NIC) each containing the gene for D10A mutated Cas9 nuclease and a unique gRNA sequence targeting CTCF. Single-cell clones were isolated and screened for CTCF depletion using the immunoassays described above. Two clones with CTCF depletion were found, and both clones showed increased CSPG4 expression.

### CUT&RUN

Cultured HeLa cells were exposed to TcdB2, and then DNA-binding sites for CTCF were isolated using CUT&RUN reagents obtained from Cell Signaling Technology (product # 86652). Antibodies used for this assay include a rabbit monoclonal antibody against CTCF (Cell Signaling Technology; product # 3418); a rabbit monoclonal antibody against H3K4me3 (Cell Signaling Technology; product # 9751); and a rabbit monoclonal isotype control (Cell Signaling Technology; product # 66362).

DNA was isolated from 2 separate CUT&RUN assays for each condition. Sequencing libraries were constructed using XGen DNA Library Prep EZ Kit (IDT), and each library was indexed in order to multiplex for sequencing. Libraries were quantified using a Qubit 4 fluorometer (Invitrogen) and checked for size and quality on a 4150 TapeStation system (Agilent Technology). Samples were normalized and pooled onto a 150 paired-end run on Illumina’s NextSeq 2000 Platform to obtain 5M reads per sample.

The FASTQ files were trimmed with Trim Galore and aligned to the human hg38 canonical genome using bowtie2 (v2.5.3) with the very sensitive end-to-end (--very-sensitive) parameter. Resulting BAM files were converted to bigWig files and normalized to counts per million (CPM) using bamCoverage. BigWig files were visualized in Integrative Genomics Viewer (IGV). To display CTCF binding throughout the genome, a matrix of gene regions *versus* signal from CTCF binding was generated in computeMatrix, and then the matrix was used to produce a heatmap with plotHeatmap.

## Data availability

RNA-Seq and CUT&RUN data were deposited at GEO with the accession numbers: GSE304120 and GSE304121.

## Conflict of interest

The authors declare that they do not have any conflicts of interest with the content of this article.
